# Supplementary material for: Long non-coding RNA produced by RNA polymerase V determines boundaries of heterochromatin
Source: eLife. 2016 Oct 25;5:e19092. doi: 10.7554/eLife.19092 (PMC5079748; doi:10.7554/eLife.19092)
Supplement: Supplementary file 1. — The table shows oligonucleotides used for locus-specific qPCR-based assays used in this study. DOI: http://dx.doi.org/10.7554/eLife.19092.016 [file elife-19092-supp1.pdf]

Table S1. Oligonucleotides Used in This Study.

Transcript	Primer orientation	Sequence
ACTIN	Forward	GAGAGATTCAGATGCCCAGAAGTC
	Reverse	TGGATTCCAGCAGCTTCCA
PolV_0290	Forward	AGCGGCCTAAATGAACATAATCCAGC
	Reverse	TCGTTGCTGGTTGTTCAAAACTGAC
PolV_0332	Forward	ATGTTTCATCTTGTTGTGGCCAAGG
	Reverse	GTTTCGACAAGGTCTTCCAAACTAAAG
PolV_0736, PolV_0737	Forward	ACCATATCCATTAATTTCGGGTTGG
	Reverse	AGTTCTGGGCACAAATATGGAACC
PolV_1057	Forward	AATTTGGTGTTGGTACATCTCAACTG
	Reverse	TTTTCACCTTCCCTTTCGAGGTGG
PolV_1468	Forward	AAAGCGATTTAGGCGGTCGACTAGG
	Reverse	ACAGTTGTCTATACGTCGCGTGAGC
PolV_1629	Forward	ATCATATCTTGCACCTCGGAAT
	Reverse	CGGGAATTTTTGCCACTAAA
PolV_1702, PolV_1703	Forward	TACCCTTGCCCTTTGTATCTTCTCC
	Reverse	GTGAGTGCCAATTTCTGCATCAAG
PolV_1818, PolV_1819	Forward	CGAAGGACGAAACTTTTTGG
	Reverse	GGTTTAAACGCAGCCAATGT
PolV_1873, PolV_1874	Forward	ATGGCCGAAATGTTGATAATGTGTAATC
	Reverse	CATGTTATGCTCAACCGGCGAC
PolV_1927, PolV_1926	Forward	GACCCATCTGCGATTCTGCGTTATG
	Reverse	GCGGATGACAGAGGGAGAACCAATC
PolV_2058	Forward	GGGCTTCCCTCTGAGTGTTT
	Reverse	CCGAAGCCCAACTAATATCG
PolV_2729	Forward	TGGCCCTTTCTCCTTCGACAACAAC
	Reverse	TTTTACATTGCAACGCACCCGTCC
PolV_2868	Forward	GACGGCACGGTTTCCTTGAATTCTC
	Reverse	GTCAAGTGGGAATGTGACACTGCGG
PolV_3151	Forward	CCTCACTCAAAGAAACGAGTTCCGAG
	Reverse	AGTGAAAGGGAGAGGAGTTGTTTGTG
PolV_3420	Forward	TTATTTTCAGGCCATAAAGAACCCAC
	Reverse	TTGTTGTAACTTGTAACTCGGACAAAG
PolV_3481	Forward	TTGTGGTCCAATTTGCTACG
	Reverse	GGCAGCAGGATATTCGGTTA
PolV_3863, PolV_3862	Forward	TCTTAATCGAACGCATGTGG
	Reverse	TGCAGCATCTGATCAACAAA
PolV_3926, PolV_3925	Forward	CGTGTCTGGTTGAGACCAAATTAGC
	Reverse	ATTAAACTCTGGAATCCGCGAGAAG
PolV_3958	Forward	TACCAACGCATCTCAAAATTGAACC
	Reverse	AAAATATTAAAGGGCGCGCTATTCG
PolV_4213	Forward	TTTGGAACAGACAATAAACCGACGC
	Reverse	AGTCTTCGACGGACTAACTACGGAC
